# Analysis of Pollution Characteristics and Emissions Reduction Measures in the Main Cotton Area of Xinjiang

**DOI:** 10.3390/ijerph20032273

**Published:** 2023-01-27

**Authors:** Chunsheng Fang, Zhuoqiong Li, Weihao Shi, Ju Wang

**Affiliations:** 1 College of New Energy and Environment, Jilin University, Changchun 130012, China; 2 Key Laboratory of Groundwater Resources and Environment, Ministry of Education, Jilin University, Changchun 130012, China; 3 Jilin Province Key Laboratory of Water Resources and Environment, Jilin University, Changchun 130012, China

**Keywords:** WRF-CMAQ, Xinjiang main cotton region, distribution characteristics of pollutants, emission reduction measures

## Abstract

With cotton production in Xinjiang increasing annually, the impact on the environment of agricultural waste produced to improve production has been reflected. This study selected Bozhou of Xinjiang, the main cotton producing region in northern Xinjiang, as the research object, and collected hourly concentration data of six pollutants from 2017 to 2021, and analyzed the spatial and temporal distribution characteristics of each pollutant. At the same time, Morlet wavelet analysis was used to further analyze the variation period of PM_2.5_ (PM particles with aerodynamic diameters less than 2.5 μm) concentration. The Weather Research and Forecasting model coupled with the Community Multiscale Air Quality (WRF-CMAQ) model was used to evaluate the emissions reduction measures for the most polluted month. The results showed that the concentration of particulate matter (PM particles with aerodynamic diameters less than 2.5 μm and 10 μm) decreased from the southern mountains to the north; moreover, the concentrations of CO (carbon monoxide), NO_2_ (nitrogen dioxide), and SO_2_ (sulfur dioxide) in the suburbs were higher than those in the urban center. The concentration of O_3_ (Ozone) was the highest in summer, while the concentrations of other pollutants were high in autumn and winter. Under the time scale of a = 13, 24, PM_2.5_ had significant periodic fluctuation. The health risk values of PM_2.5_ and PM_10_ in this study were within the scope of the United States Environmental Protection Agency (USEPA) criteria, but it is still necessary to keep a close watch on them. In the context of emissions reduction measures, agricultural sources reduced by 20%, residential sources by 40%, industrial sources by 20%, and transportation sources by 20%; no change in the power source remains. Under these conditions, the daily average value of each pollutant met the first level of the national ambient air quality standard. The research results provide a reference for the local government to formulate heavy pollution emissions reduction policies.

## 1. Introduction

The Xinjiang Uygur Autonomous Region in the northwest of China is the largest high-quality cotton producing province in China, and the cotton output has increased rapidly in the past decade [1]. The cotton yield in Xinjiang in 2021 was 5.129 million tons, accounting for 89.5% of the total national output [2]. Borla Mongol Autonomous Prefecture (hereinafter referred to as Borla) is located at 79°53′–83°53′ E, 44°02′–45°23′ N, in the hinterland of Eurasia. The mountains in the west, north, and south belong to the continental arid climate of the northern temperate zone. As the main cotton-producing area in northern Xinjiang, Bozhou has 91,100 hectares of cotton. However, excessive use of pesticides and straw burning were the most prominent problems in the cotton planting. Some pesticides entered the atmosphere in the form of ammonia volatilization pollutants, which generate greenhouse gas emissions, O_3_, and acid rain hazards [3,4]. Under the stable layer of low temperature and calm wind in autumn and winter, the burning of crop straw causes the concentration of particulate matter to exceed the standard in a specific time period, which will cause harm to human health and the ecological environment [5,6]. Particulate matter is the most harmful to human health [7]. According to epidemiological studies, particles enter blood circulation through the alveoli, cause respiratory diseases, and increase mortality and cancer risk [8,9,10,11].

Therefore, it is particularly important to take emissions reduction measures promptly for heavy pollution. At present, the research on emissions reduction effects is mainly based on field observations and air quality model simulations. The observation method has some limitations. For example, it is impossible to predict and evaluate the measures that have not been implemented, and it is impossible to exclude the impact of meteorological conditions [12]. W. He et al. [13] used the CMAQ model to analyze the impact of vehicle emission control measures, and combined policy scenarios on ambient air quality. Chen et al. [14] calculated the contribution rate of emissions reduction to PM_2.5_ using KZ filtering and the WRF-CMAQ model. The results showed that the controlling man-made contribution rate declined about 80% in Beijing, and emissions reduction was crucial to the improvement in air quality in Beijing from 2013 to 2017. Yu et al. [15] used the WRF-CMAQ model to assess the impact of meteorological and emission control measures on the improvement in air quality in Guangzhou and assessed the contributions of various sources to emissions reduction.

This study selected Bozhou, the main cotton producing region in northern Xinjiang as the research object, and collected hourly data of six kinds of pollutants from 2017 to 2021. We analyzed the spatial and temporal distribution characteristics of each pollutant. At the same time, Morlet wavelet analysis was used to further analyze the change period of PM_2.5_ concentration. Then, we conducted a health risk assessment. Finally, the WRF-CMAQ model was used to evaluate the impact of emissions reduction measures on air quality. This study expects to provide some basis for improving the environmental air quality control and emissions reduction measures of heavily polluted weather in Bozhou. It could also provide a case reference for the study of regional pollutant change characteristics.

## 2. Materials and Methods

### 2.1. Study Area and Data Source

**Figure 1:**
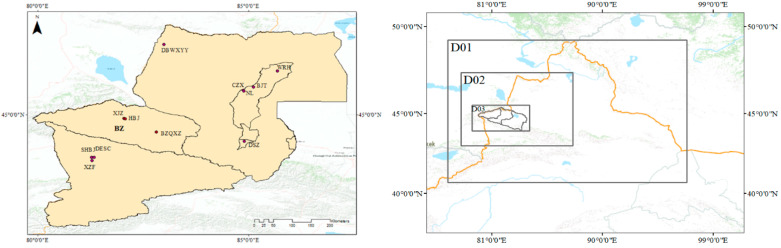
National environmental quality monitoring stations, weather stations and modeling domains.

The monitoring data came from the stations of the China National Environmental Monitoring Network (https://www.aqistudy.cn/historydata/) (accessed on 5 October 2022). This monitoring website has provided the air quality index (AQI) at different locations in China since 2014, as well as the daily average concentration data of PM_2.5_, PM_10_, SO_2_, NO_2_, and CO. The two environmental monitoring stations and one meteorological station in Bozhou are the Western Suburb of Bole City (XJQ), the Municipal Environmental Protection Bureau (HBJ), and the meteorological station (BZQXZ), respectively. All meteorological data were from the China Meteorological Data Service Center (http://data.cma.cn) (accessed on 6 October 2022). Figure 1 shows the locations of urban monitoring stations and meteorological stations near Bozhou.

### 2.2. Model

Joint Weather Research and Prediction Model (WRF) version V4.1.2 and Community Multi-Scale Air Quality Simulation System (CMAQ) version 5.3.2 were used in this study. Driven by the meteorological field generated by the WRF model, the CMAQ model is used to simulate the different physical and chemical processes of gaseous pollutant and particulate matter (PM) composition. The meteorological data used in the WRF-CMAQ model adopt the Environmental Prediction Final Analysis (NCEP-FNL) reanalysis data. The emissions inventory uses the high-resolution anthropogenic Emissions MEIC Inventory (2017) developed by Tsinghua University. The MEIC is an emission inventory model framework based on bottom-up technology. The resolution is 0.25° × 0.25°, and contains emissions information from five different sectors, namely the power, industry, residential, transport, and agriculture sectors.

Figure 1 shows the study area and three nested areas. WRF-CMAQ simulation was conducted on three nested domains. Domain 1 covered the northern Xinjiang region of Xinjiang Province, with a grid resolution of 27 km × 27 km; domain 2 covered Ili, Karamay, and Bozhou in Xinjiang, with a grid resolution of 9 km × 9 km; and domain 3 covered Bozhou City, and the grid resolution was 3 km × 3 km. In WRF simulation, the vertical grid structure consisted of 29 layers, with a center longitude and latitude of 84.69° E and 41.89° N. The simulation period was January, April, July, and October 2017. The CMAQ model was configured using the AER06 aerosol module and CB06 gaseous chemical mechanism. In order to eliminate the impact of initial conditions, the model ran 5 days in advance.

### 2.3. Wavelet Analysis of Time Series

In order to further explore the time series of pollutant concentration changes and reveal different change cycles in the whole time series, this study used wavelet analysis [16] to analyze the daily average PM_2.5_ concentration data of Bozhou in 2017.

The wavelet basis functions are expressed in Equation (1)

(1) $$\psi_{a,b}\left(t\right)={\left|a\right|}^{-1/2}\psi \left(\frac{t-b}{a}\right)$$

Among them, *a, b*,∈ *R*, and *a* ≠ 0. In the above equation, a represents the time scale, mainly used to show the length of the wavelet period; *b* is the translation parameter, which mainly shows the passage of wavelet in time.

The continuous wavelet transform (CWT) is show in Equation (2)

(2) $$W_{f}\left(a,b\right)={\left|a\right|}^{-1/2}\int_{R}f\left(t\right)\bar{\psi }\left(\frac{t-b}{a}\right)dt$$

In the above formula, $W_{f}\left(a,b\right)$ is the wavelet transform coefficient; f(t) is a finite signal; and $\bar{\psi }\left(\frac{t-b}{a}\right)$ is the complex conjugate function of $\left(\frac{t-b}{a}\right)$.

The wavelet square difference is mainly obtained by integrating the square value of the wavelet coefficient. The formula is show in Equation (3)

(3) $$\mathrm{Var}\left(a\right)=\int_{-\infty }^{\infty }{\left|W_{f}\left(a,b\right)\right|}^{2}db$$

The transform curve of wavelet squares with the time scale parameter a is the wavelet squares graph, and the scale a corresponding to its maximum value is the main period of time series variation.

### 2.4. Health Risk Assessment

The health risk assessment of pollutants with non-carcinogenic effects was used to assess the health risk of particulate matter in the urban atmospheric environment [17], the calculation formula is as follows:

(4) $$R=\frac{C}{Rfc}\times 10^{6}\;$$

In the above formula, *R* represents the health risk of a certain hazard, *C* is the monthly average concentration of PM_2.5_ or PM_10_, and *Rfc* is the reference concentration of the substance to be evaluated. The daily average concentration of fine particles in national ambient air quality standard is the RFC value [18].

## 3. Results and Discussion

This section is divided by subheadings. It provides a concise and precise description of the experimental results, their interpretation, as well as the experimental conclusions that were drawn.

### 3.1. Difference in Spatial Distributions of Pollutant Concentrations

**Figure 2:**
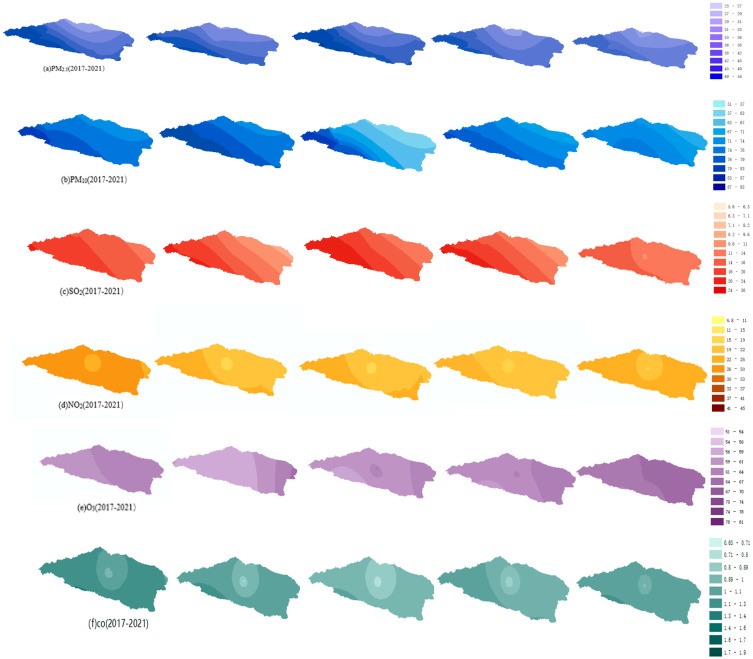
Spatial distribution map of six pollutants in Bozhou from 2017 to 2021 (CO units: mg/m^3^, others are μg/m^3^).

Two ambient air quality monitoring stations were located in the center of Bole. Due to the small number of points, this study calculated the spatial concentration distribution of pollutants using the annual average concentration of each station, in combination with the air quality monitoring stations in Yili, Karamay, and Tacheng nearby Bozhou. Figure 2 shows the spatial distribution characteristics of pollutants in Bozhou from 2017 to 2021. The pollutant concentration shows a decreasing trend from 2017 to 2021, and the main pollutant was particulate matter. The minimum average concentrations of PM_2.5_ and PM_10_ in 2020 were 21.95 μg/m^3^ and 59.41 μg/m^3^. Bozhou city is part of the Tianshan fold belt, surrounded by mountains on three sides in the northwest and south. Due to the rapid heat emissions of the hillside, the original warm air at the foot of the mountain is lifted by sinking cold air; the temperature inversion phenomenon is common in winter, which leads to high atmospheric pollutants [19]. The burning of crop straw in the autumn and winter seasons also leads to an increase in particulate matter concentration. The concentration of particulate matter decreased from south to north. Since 2017, Bozhou has implemented the air pollution prevention plan, promoted the pollution control of coal-fired boilers, and promoted the adjustment of energy structure [20]. The concentration of particulate matter has decreased in recent years.

According to Ghosh D. et al. [21], the seasonal variation in surface CO concentration is high in the winter, and the CO concentration in the central urban area is higher than that in the suburb. The results of this study are consistent with those of others. This distribution is due to the fact that traffic flow in central urban areas is higher than that in marginal urban areas. Most CO emissions come from fuel-burning private cars, and traffic flow also increases the concentration of NO_2_ to a certain extent. The average concentration of CO in 2021 was lower than that in 2017. The concentration of O_3_ increased from west to east because there was a large area of cotton planting east of the city, which released many precursors of O_3_, such as VOCs, which are conducive to the generation of O_3_ [22,23]. Sayram Lake is located in the southwest suburb of Bozhou, which is a natural scenic spot, so the concentration of O_3_ is low all year round.

The spatial concentration of SO_2_ was consistent with that of CO and NO_2_, and the concentration in the suburbs was higher than that in the center of the city. This may be because the cotton planting area and coal-fired boilers are located in the suburbs of the city, resulting in higher concentrations of SO_2_ in the atmosphere in remote areas closer to the west. The concentration of SO_2_ and NO_2_ was high. In recent years, in Bozhou, factory fuel has been gradually replaced by clean energy, and the soot generated is desulfurized before chimney discharge, which also results in decreased SO_2_ concentration discharged from the factory chimney to a large extent, so the SO_2_ concentration in the atmospheric troposphere decreases year by year.

### 3.2. Temporal Variation Characteristics of Pollutants

#### 3.2.1. Annual and Monthly Variation Characteristics

**Figure 3:**
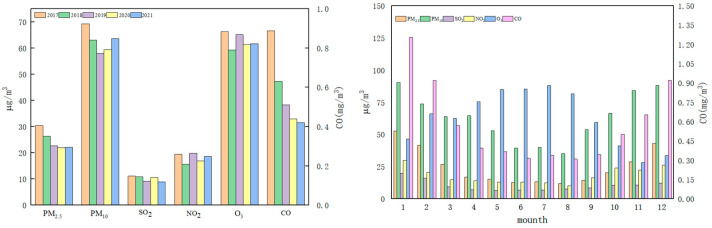
Variation chart of the concentrations of six pollutants, and average monthly concentrations of pollutants in Bozhou from 2017 to 2021.

Figure 3 shows the annual average concentration changes in pollutants, and the average monthly concentration changes of pollutants in Bozhou City, from 2017 to 2021. Overall, in the five years from 2017 to 2021, the concentrations of all pollutants showed a downward trend. Since 2017, Bozhou has conducted an air pollution prevention action plan, accelerated the elimination of coal-fired boilers, and promoted the upgrading of industrial enterprises. Therefore, the concentration of most pollutants declined in 2017–2021.

The monthly average concentration changes in six pollutants in Bozhou City from 2017 to 2021 are shown in Figure 3. According to the local temperature, cotton in Xinjiang is planted around May every year. When planting cotton, local farmers apply base fertilizer to the cultivated land, which mainly includes phosphorus fertilizer, urea, and nitrogen fertilizer. Therefore, the concentrations of NO_2_ and SO_2_ increased from May to June. In the process of cotton growth, a quantity of pesticides such as insecticide and defoliant are applied until September and October for picking and purchasing. However, a large amount of chemical fertilizer is applied to the cotton planting area. This process leads to a considerable part of nitrogen fertilizer entering the atmosphere in the form of nitrification–denitrification and ammonia volatilization [24]. Therefore, the concentrations of NO_2_ and SO_2_ also show an upward trend from July and September. These pollutants are easy to be oxidized, which leads to the destruction of the ozone layer on the one hand, and an important source of acid deposition on the other hand. Acid rain causes harm to crops, buildings, and human health [25]. September to October is the cotton picking and purchasing season in Xinjiang. The picking process and the large-scale use of harvesting machines leads to a temporary increase in the concentration of PM_2.5_ and PM_10_ in the air. Bozhou is located in the north of Xinjiang, where the landform is mainly arid desert. It is one of the sources of large particles of PM_10_, close to the source of Siberian dust storms, and has frequent sandstorms in spring and autumn [26]. 

#### 3.2.2. Seasonal Hourly Mean Change

**Figure 4:**
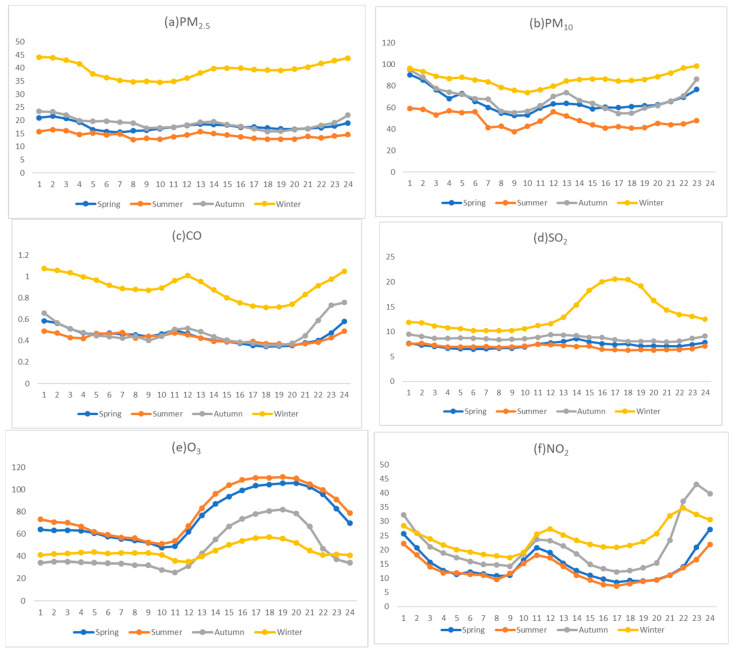
Seasonal variations in the concentrations of six pollutants in Bozhou (CO units: mg/m^3^, others are μg/m^3^).

It can be seen from the above analysis that the spatial distributions of pollutants in 2017–2021 are different, so the seasonal hourly average concentration of pollutants in 2017–2021 was selected to analyze the seasonal hourly change in pollutants. As shown in the Figure 4, the air pollutants in Bozhou City had obvious change characteristics. The seasonal average concentrations of PM_2.5_, PM_10_, SO_2_, NO_2_, and CO were the highest in winter, and their mass concentrations were 44.17 μg/m^3^, 99.13 μg/m^3^, 20.60 μg/m^3^, 34.72 μg/m^3^, and 1.07 mg/m^3^, respectively. Spring and autumn were next, and summer was the lowest. These results are consistent with those of Wang li et al. [27]. According to the hourly average concentration values, SO_2_ showed obvious diurnal variation characteristics in winter, reaching the highest value of 20.60 μg/m^3^ at 17:00. This was because the average temperature in the Bozhou area is the lowest in the year in winter—especially at night when the heating intensity increases, the use of coal increases—and because of the gradual accumulations of NO, NO_2_, VOC, and CO in winter. Thus, the emissions of pollutants related to it increased at night, leading to a reduction in air quality. The proportions of PM_2.5_ and PM_10_ were relatively high in winter. Coal burning and crop straw burning in winter had a relatively high contribution to particulate matter. Moreover, the geographical location of Bozhou, under the stable stratification of low temperature and calm winds in winter, was more conducive to the accumulation of pollutants that endanger human health. The average mass concentration of O_3_ in spring and summer was significantly higher than that in autumn and winter, and showed obvious diurnal variation characteristics. This is consistent with the research results of Tui et al. [28]. During the period from 11:00 to 14:00 in summer, the concentration of O_3_ rose rapidly, reaching a peak of 111 μg/m^3^ at 18:00; then, with the weakening of solar radiation, the temperature decreased, and so did the concentration of O_3_. The higher temperature in summer, accompanied by strong solar radiation and long daily sunshine, increased the ozone concentration, while low temperatures in autumn and winter, weak solar radiation, and no sunshine time inhibited the photochemical reaction, leading to a reduction in O_3_ concentration [29,30]. 

### 3.3. PM_2.5_ Concentration Variation Cycle Study

In this study, the time series of PM_2.5_ daily average concentrations in 2021 was selected for Morlet wavelet analysis, to further analyze the change cycle and future trends in pollutant concentrations. The distribution of PM_2.5_ concentrations in the time domain and the regularity of periodic changes in each time scale can be seen from the contour map of the real part of the wavelet coefficients. The main period of concentration change can be seen from the wavelet variance diagram of the wavelet coefficients of the original time series of PM_2.5_ daily average concentrations.

**Figure 5:**
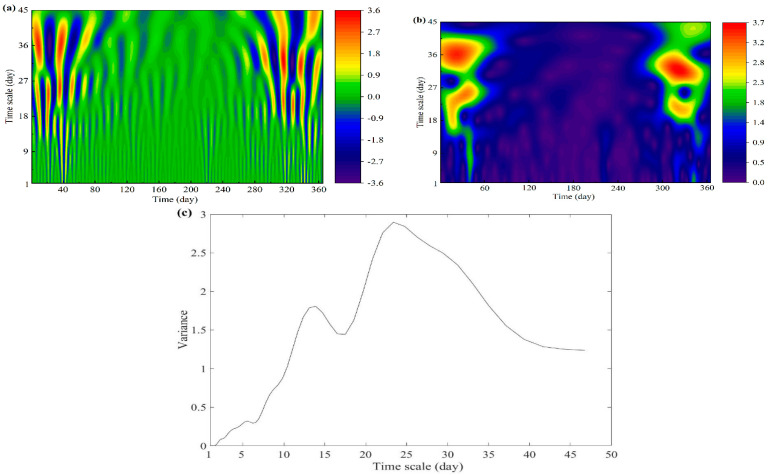
Wavelet coefficient diagram (**a**); wavelet energy diagram (**b**); wavelet variance diagram (**c**).

Figure 5 shows the multi-time scale characteristics of PM_2.5_ daily average concentration values. On the whole, the annual concentration values showed a trend of alternating cycle changes, and the distribution intensity on each time scale showed a certain difference. As the time scale increased, the pollutant concentration change cycle continued to extend, and the fluctuation energy continued to increase. From the contour map of wavelet coefficients, it was found that the periodic variation in PM_2.5_ concentration was regional, on the scale of 7–17 days and 21–37 days. The rule of contour and color change in the early and late stages were obvious, while the medium-term periodicity was weak.

It can be seen from the wavelet variance chart (Figure c) that there are three peaks, two of which are more obvious; that is, when the time scale of a = 13 and 24, the wavelet variance had a maximum, which is consistent with the rule shown in the contour map. When the time scale of a = 24, the time scale corresponded to the maximum peak, and the energy fluctuation in the wavelet energy map was the largest, which was the first main cycle of PM_2.5_ concentration change, showing the largest time series fluctuation energy and the strongest periodic oscillation.

### 3.4. Health Risk Assessment

**Figure 6:**
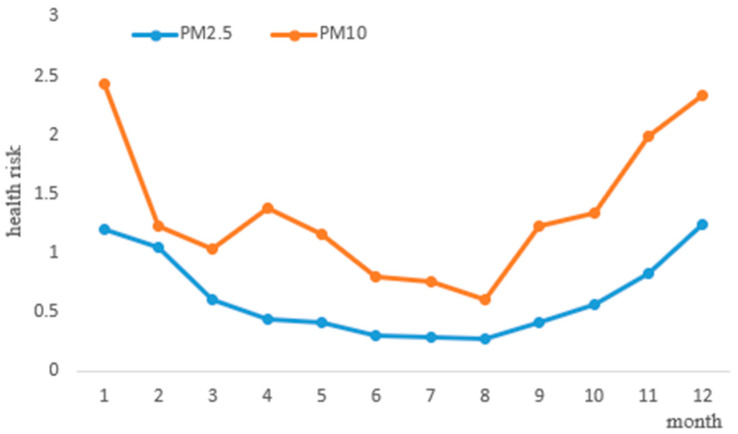
The health risk assessment values of PM_2.5_ and PM_10_.

Based on the health risk assessment and environmental monitoring data, we calculated the health risk values of PM_2.5_ and PM_10_ in Bozhou in 2021. The results are shown in Figure 6. The health risk value of PM_2.5_ in the environment was 0.27 × 10^−6^ − 1.24 × 10^−6^, the PM_10_ health risk value was 0.61 × 10^−6^ − 2.33 × 10^−6^. The PM_10_ health risk value was generally higher than that for PM_2.5_. In addition, the health risk values of both from January to December showed a trend of decreasing first, and then increasing. At the same time, the high values both appeared in January and December. The PM_10_ health risk value increased slightly in April. However, both were within the acceptable risk range established by the USEPA (1.00 × 10^−6^~1.00 × 10^−4^). According to epidemiological studies, particulate matter can enter blood circulation through the alveoli, cause respiratory diseases, and increase mortality and cancer risk [31,32]. The health risk values of PM_2.5_ and PM_10_ in this study were within the scope of the USEPA, but it is still necessary to monitor these closely. The government should take emissions reduction measures for serious air pollution and high health risk values for January and December.

### 3.5. Model Validation

#### 3.5.1. Verification of WRF Meteorological Simulation Results

**Figure 7:**
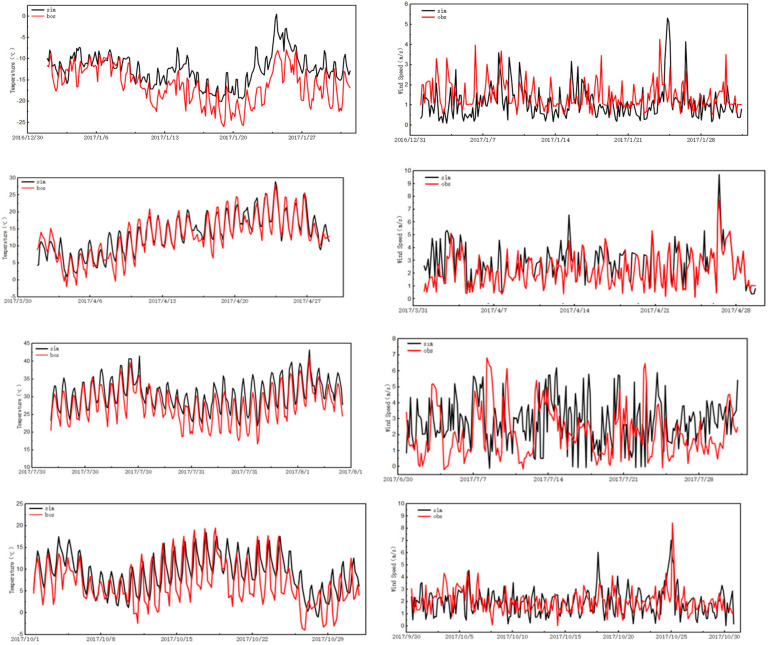
Time series of temperature and wind speed in Bozhou in January, April, July, and October 2017.

Since the meteorological input data provided by the WRF model directly affect the uncertainty of CMAQ simulation results, the WRF model was first verified. The typical seasonal months of January, April, July, and October in 2017 were selected for the simulation period, and T2 and WS10 were verified. The model performance was evaluated according to three statistical indicators: correlation coefficient (R), mean score deviation (MFB), and mean score error (MFE). The comparison of simulation results and monitoring results is shown in Table 1 and Figure 7. R conforms to the critical value of the correlation coefficient; MFB and MFE results conform to the standards proposed by the US Environmental Protection Agency, and are slightly higher than the standards in July. The model performance results showed that the WRF model could simulate temperature and wind speed well. The missing data of the meteorological station were less than 1%. In general, the simulation results from real weather conditions provided reliable weather input for the CMAQ.

#### 3.5.2. Verification of CMAQ Model Results

**Figure 8:**
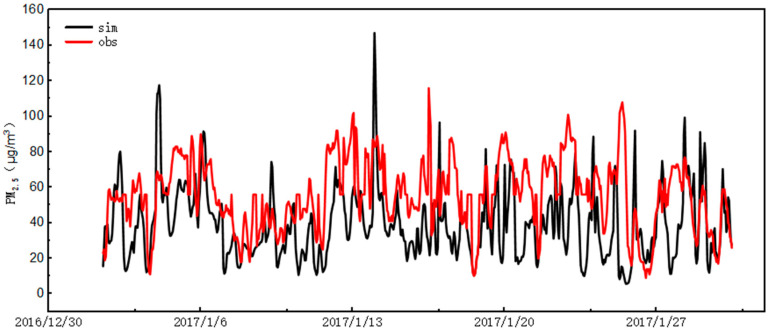
Time series of Bozhou PM_2.5_ in January 2017.

Figure 8 shows the comparison results of model simulation and monitoring results in Bozhou City from 2 to 30 January 2017. Table 2 summarizes the statistical results of the PM_2.5_ simulation performance, including correlation coefficient (R), mean fractional deviation (MFB), and mean fractional error (MFE). The MFB and MFE results met the standards proposed by the US Environmental Protection Agency. The missing data of the air quality monitoring station were less than 10%. In general, the model captured the time variation characteristics of the PM_2.5_ concentration at the observation station. However, the model sometimes underestimated the PM_2.5_ concentration, and the simulation value reached 65% of the monitoring value. This is because part of the first and second nested grids were outside the Chinese border, where there were no specific emission inventory data, leading to low simulation results. The verification results showed that the CMAQ model could accurately verify the change in PM_2.5_ concentration of pollutants in January.

### 3.6. Assessment of Emission Reduction Measures

According to the previous analysis, the average annual concentration of pollutants in Bozhou City reached GB3095 standard in 2017, but in January, the most seriously polluted month, the daily average concentration was still not up to the standard due to crop straw burning, winter heating, and dust weather. Therefore, this study takes emission reduction measures for January, the most polluted month in 2017, and the specific reduction ratio is shown in the Table 3. In the BAS scenario, agricultural sources are reduced by 10%, residential sources by 30%, industrial sources by 10%, transportation sources by 10%, and power sources remain unchanged. In the BET optimization scenario, agricultural sources are reduced by 20%, residential sources by 40%, industrial sources by 20%, and transportation sources by 20%. The power source remains unchanged. To explore the impact of various emissions reduction scenarios on the air quality concentration of Bozhou in January 2017.

**Figure 9:**
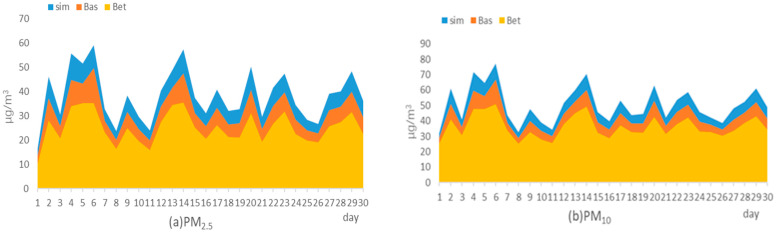
Variation chart of daily average of Bozhou PM_2.5_ and PM_10_ emission reduction scenarios and simulations in January 2017.

In January of the model simulation, particulate matter was the main pollutant. There were 57% of the days with a PM_2.5_ concentration greater than 35 μg/m^3^, 43% of PM_10_ days were greater than 50 μg/m^3^, wherein the maximum daily concentration of PM_2.5_ was 59.17 μg/m^3^; the maximum daily concentration of PM_10_ was 77.31 μg/m^3^. The concentration of other pollutants met the national Class 1 standard. As shown in the Figure 9, in the BAS scenario, after reducing various sources, PM_2.5_ and PM_10_ decreased by 14–19% and 10–17%, respectively. A total of 30% of the days still exceeded the standard, and the highest daily concentrations of PM_2.5_ and PM_10_ were 49.61 μg/m^3^ and 66.49 μg/m^3^, respectively, which were still higher than the national pollutant concentration level 1 standard. SO_2_ decreased by 15–30%, NO_2_ by 4–14%, and CO by 16–21%, among which, the concentration of O_3_ was negatively correlated with the concentration of PM_2.5_, with a slight increase of 3–16%. The concentration of O_3_ met the national standard. In the BET scenario, the daily concentrations of PM_2.5_ and PM_10_ were less than 35 μg/m^3^ and 50 μg/m^3^, respectively, in which PM_2.5_ and PM_10_ decreased by 28–40% and 20–34%, respectively. The reduction ratio of SO_2_ was 30–54%, that of NO_2_ was 10–28%, that of CO was 32–41%, and that of O_3_ was 10–30%. Under the BET scenario, all pollutants met the first level of the national ambient air quality standard.

## 4. Limitation and Future Direction

There are also some limitations in this study. Firstly, we only used emission information from five sectors. The inventory, through continuous in-depth study in the future, will include more emissions information, such as biomass fuels (straw). Secondly, the setting of the time allocation coefficient of the emission inventory in this study was still not perfect. In future research, different time allocation coefficients will be set for different months in order to further study regional composite pollution. In the future, we will further refine the setting of emissions reduction plans, set up multiple emissions reduction scenarios for different industries, different regions, and different times, and evaluate the emissions reduction effect of emissions reduction scenarios.

## 5. Conclusions

This study analyzed the change characteristics of air pollutants in Bozhou, and further analyzed the change period of PM_2.5_ concentration using Morlet wavelet analysis. The WRF-CMAQ model was used to take emissions reduction measures in January 2017, when Bozhou was heavily polluted. The results are as follows.

The concentration of particulate matter decreased from the southern mountains to the north, and the concentrations of CO, NO_2_, and SO_2_ in the suburbs were higher than those in the urban center. The pollutant concentration showed a downward trend from 2017 to 2021. The annual average concentrations of all kinds of pollutants in 2021 were the lowest. The heavy application of pesticides and fertilizers in cotton planting areas led to a small increase in SO_2_ and NO_2_ concentrations from May to October. The concentration of O_3_ in summer was the highest, while the concentration of other pollutants was high in autumn and winter, and low in spring and summer. Under the time scale of a = 13, 24, the PM_2.5_ had significant periodic fluctuation. The health risk values of PM_2.5_ and PM_10_ in this study were within the scope of United States Environmental Protection Agency (USEPA) criteria, but they still need to be monitored closely. In January 2017, emissions reduction measures were taken for the most seriously polluted areas. Under the BAS scenario, the pollutant concentrations of PM_2.5_ and PM_10_ still exceeded the standard for 30% of the days. Under the BET scenario, all pollutant concentrations met the first level of the national ambient air quality standard. Under the emissions reduction measures, air quality significantly improved, and the research results can provide a reference for the local government to formulate heavy pollution emissions reduction policies.

## Figures and Tables

**Table 1:** Statistical indicators for WRF model evaluation in Bozhou in January, April, July, and October 2017.

		January	April	July	October
T2	R	0.77	0.93	0.86	0.79
	MFB	−14.8%	6%	9%	−13.9%
	MFE	−16%	12%	13%	−15%
WS10	R	0.29	0.65	0.12	0.27
	MFB	15%	−8.78%	16%	14%
	MFE	34.8%	20%	38%	35%

**Table 2:** Statistical indicators of Bozhou CMAQ model evaluation in January 2017.

	R	MFB	MFE
PM_2.5_	0.35	−21%	28%

**Table 3:** Emissions reduction scenarios in Bozhou.

	AG	AR	IN	PP	TR
BAS	10%	30%	10%		10%
BET	20%	40%	20%		20%

## Data Availability

Not applicable.
